# Cellular phenotypic transitions in diabetic nephropathy: An update

**DOI:** 10.3389/fphar.2022.1038073

**Published:** 2022-11-02

**Authors:** Yiling Cao, Ji-Hong Lin, Hans-Peter Hammes, Chun Zhang

**Affiliations:** ^1^ Department of Nephrology, Union Hospital, Tongji Medical College, Huazhong University of Science and Technology, Wuhan, China; ^2^ 5th Medical Department, Medical Faculty Mannheim, University of Heidelberg, Mannheim, Germany

**Keywords:** diabetic nephropathy, phenotypic transition, fibrosis, EMT, EndoMT, signaling pathway

## Abstract

Diabetic nephropathy (DN) is a major cause of morbidity and mortality in diabetes and is the most common cause of end stage renal disease (ESRD). Renal fibrosis is the final pathological change in DN. It is widely believed that cellular phenotypic switching is the cause of renal fibrosis in diabetic nephropathy. Several types of kidney cells undergo activation and differentiation and become reprogrammed to express markers of mesenchymal cells or podocyte-like cells. However, the development of targeted therapy for DN has not yet been identified. Here, we discussed the pathophysiologic changes of DN and delineated the possible origins that contribute to myofibroblasts and podocytes through phenotypic transitions. We also highlight the molecular signaling pathways involved in the phenotypic transition, which would provide valuable information for the activation of phenotypic switching and designing effective therapies for DN.

## 1 Introduction

With rapid urbanization and significant changes in lifestyles, diabetes mellitus (DM) rapidly become a global health burden, which impacts approximately 9% of the population worldwide (Domingo-Lopez et al., 2022). The disease is characterized by chronic hyperglycemia due to inadequate insulin secretion or reduced insulin sensitivity (American Diabetes Association Professional Practice, 2022). The morbidity and mortality of diabetes are mostly related to the development of diabetes-associated complications affecting the kidney and other organ systems. Diabetic nephropathy (DN) usually occurs in patients with DM without long-term adequate glycemic control (Lin et al., 2018). About one-third of patients with type 1 DM and half with type 2 DM will develop chronic kidney disease, which is clinically defined by the presence of impaired renal function or elevated urinary albumin excretion, or both (Akhtar et al., 2020). DN is a chronic progressive disorder and is the leading cause of end stage kidney disease in the United States and most developed countries, representing in some parts of the world more than 50% of subjects requiring kidney replacement therapy (Collins et al., 2014; Umanath and Lewis, 2018). The kidneys undergo several changes and result in a clinical presentation including persistent albuminuria, hypertension, and progressive reductions in glomerular filtration rate (GFR) (Umanath and Lewis, 2018; Sugahara et al., 2021).

The most typical morphological change of DN is the excessive deposition of extracellular matrix (ECM) proteins in the mesangium and basement membrane of the glomerulus and the renal tubulointerstitium, leading to glomerulosclerosis and interstitial fibrosis (Mason and Wahab, 2003). Renal fibrosis is the final pathological change in DN (Humphreys, 2018). It is widely accepted that the activated myofibroblasts are the principal effector cells that are responsible for the excess deposition of interstitial ECM under pathological conditions, but their origin in the kidney remains uncertain (Humphreys, 2018; Yuan et al., 2019). The phenotype switching of fibroblasts to secrete and remodel the extracellular matrix is one of the origins of myofibroblasts. However, emerging evidence suggests that diabetes also alters the phenotype of normal, non-fibroblast kidney cells, such as podocytes, mesangial cells, and tubular epithelial cells. As a result, differentiated kidney cells become reprogrammed to secrete and accumulate extracellular matrix (Simonson, 2007). Moreover, major advances have been achieved in parietal epithelial cells (PECs) in recent years. PECs have been considered to have the capacity to transdifferentiate toward podocytes in DN, which may serve as a potential therapeutic target to replace podocytes. Identification of the mechanisms underlying these processes is necessary for the development of future therapies. This review aims to further understand the transdifferentiation processes in the DN and provide ideas for designing new prevention and therapeutics. The regulation of phenotypic transitions in the development process of DN should yield productive clues for therapies.

## 2 Pathophysiology of DN

DN usually manifests a clinical syndrome including increased urine albumin excretion (>300 mg/day), persistent reduction in GFR, and increased blood pressure (Sugahara et al., 2021). The cause of DN is the high blood glucose that destroys the blood vessels in the kidneys resulting in its dysfunction (Gajjala et al., 2015). The pathogenesis of diabetic nephropathy has been deeply investigated, and the roles of various mechanisms have been described, which include the effect of high glucose, inflammation, oxidative stress, renin-angiotensin system activation, and endoplasmic reticulum stress, etc (Rask-Madsen and King, 2013; Gujarati et al., 2021). These changes lead to various cellular responses, the expression of secretory factors and extracellular matrix accumulation (Maezawa et al., 2015). Hyperglycemia can cause several morphological changes that involve all sections of the kidney. Early alterations include the development of glomerular hyperfiltration and hypertrophy (DeFronzo et al., 2021). As the disease progresses, ECM, such as collagen I and IV and fibronectin (FN) accumulation, mesangial cells proliferation, podocyte foot process effacement and number decrease, basement membrane thickening, tubular atrophy and peritubular capillary rarefaction, culminating in interstitial fibrosis and glomerulosclerosis. Clinical manifestations show increased urinary albumin excretion, progressive loss of renal function and ultimately kidney failure (Carew et al., 2012; Umanath and Lewis, 2018).

The major structural abnormality of the glomerulus is mesangial matrix expansion. This is initially due to increased mesangial cell proliferation and matrix deposition (Thomas and Ford Versypt, 2022). As the disease progresses, matrix accumulation is the predominant mesangial change. Glomerular basement membrane (GBM) is diffuse thickening and filled with extracellular matrix components, which gradually occludes the glomerular capillaries, thus reducing the area available for glomerular filtration and leading to proteinuria or macromolecular leakiness (Fioretto and Mauer, 2007; Vasanth Rao et al., 2019; Akhtar et al., 2020). Diffuse GBM thickening has been demonstrated in almost all diabetic patients (Zhang et al., 2018a). Epithelial podocyte foot processes, together with the fenestrated endothelium and the GBM form the glomerular filtration barrier (Zhou and Liu, 2015). Podocytes wrap the glomerular capillaries and are attached to the GBM. Podocyte detachment and foot process effacement owing to cytoskeletal changes and podocyte phenotypic alterations is the principal cause of albuminuria in DN (Li et al., 2008; Wang et al., 2019). Extensive podocyte damage will eventually result in podocyte loss and a decrease in density in the development of DN (Tharaux and Huber, 2012). During the phenotypic transition, this architecture of podocytes is disrupted and the actin cytoskeleton is redistributed, which induced structural alterations to the slit diaphragm and foot process effacement (Garg and Holzman, 2012). The podocyte detachment causes the areas of bare GBM. The exposed GBM region would subsequently attach to the PECs and the Bowman’s capsule, which along with ECM deposition subsequently leads to glomerulosclerosis (Li et al., 2007; Mora-Fernandez et al., 2014).

Tubulointerstitial fibrosis is a consistent feature of DN, not just the consequence of glomerular injury (Tonolo and Cherchi, 2014). The pathological changes show tubular hypertrophy, increased apoptosis, thickening of the tubular basement membrane, interstitial inflammatory cell infiltration and finally tubular atrophy and tubulointerstitial fibrosis (Liu et al., 2019). Tubulointerstitial fibrosis features interstitial matrix deposition, inflammation, fibroblast activation, microvascular rarefaction, and tubular cell loss (Peng et al., 2022). Hyperglycemia causes renal tubular epithelial cells to transform into mesenchymal cells, leading to increased expression of FN and α-smooth muscle actin (α-SMA) (Hung et al., 2021). Injured tubular epithelial cells (TECs) could also release many effectors like inflammatory cytokines and chemokines on surrounding cells such as fibroblasts, causing them to transform into myofibroblasts and produce ECM components. The progression of glomerulosclerosis and tubulointerstitial fibrosis ultimately causes a deterioration of renal dysfunction in DN.

## 3 Phenotypic transitions in DN

Hyperglycemia causes long-term renal injury and increased deposition of ECM, which constitute the main drivers of renal fibrosis. Although many types of cells can produce ECM, it is widely accepted that myofibroblasts play a major role in the synthesis and secretion of ECM under pathological conditions (Duffield, 2014). A recent study demonstrated that the majority of scar tissue in human and mouse kidney fibrosis originates from platelet-derived growth factor receptor α (Pdgfrα+)/platelet-derived growth factor receptor alpha β (Pdgfrβ+) dual-positive fibroblasts and myofibroblasts (Kuppe et al., 2021). Myofibroblast is characterized by its signature protein α-SMA and has a greater capacity for generating collagen fibers (Liu, 2011). However, the origin of myofibroblasts in DN remains controversial (Loeffler and Wolf, 2015). With much research in the past years focused on the origin of myofibroblasts, there are several known sources of myofibroblasts, including resident mesenchymal cells (fibroblasts and pericytes), the transition from resident endothelial cells (ECs) or TECs through endothelial-mesenchymal transition (EndoMT) and epithelial-mesenchymal transition (EMT) (Schrimpf et al., 2012; Srivastava et al., 2019). A study in 2013 used multiple genetically engineered mice to track the source of myofibroblasts. It was found that the source of myofibroblasts is 50% arising from the proliferation of resident fibroblasts, 35% through differentiation from bone marrow, 5% from EMT, and 10% from EndoMT (LeBleu et al., 2013). A new study employed single-cell RNA-seq revealing pericytes and fibroblasts as the major cellular sources of myofibroblasts, while tubular epithelia, endothelium and monocytes only exhibited minor ECM expression (Kuppe et al., 2021).

EMT, reactivated in wound healing, fibrosis and cancer progression, is a complex set of phenotypical changes induced in epithelial cells that lose the hallmark epithelial characteristics including cell-cell basement membrane contacts, structural polarity and reorganizing their cytoskeleton, which changes the signaling program that defines cell shape and reprogrammes gene expression (Lamouille et al., 2014). EMT has been classified into three distinct classes: Type I EMT is associated with embryogenesis and organ development; type II EMT is induced in the context of inflammation and fibrosis, and type III EMT is associated with cancer progression (Zeisberg and Neilson, 2009; Carew et al., 2012). Type II EMT is commonly defined as the ability of epithelial cells to undergo dedifferentiation in fibrosis. Overexpression of many EMT inducers increases the expression of important transcription factors like Snail family transcriptional repressor (Snail), Twist family BHLH Transcription Factor (Twist) and Zinc finger E-Box binding homeobox (ZEB) that regulate EMT (Barrallo-Gimeno and Nieto, 2005; Feng et al., 2022).

EndoMT is a subtype of EMT. ECs compose the internal surface of the vessels and play an essential role in vascular homeostasis. EndoMT plays an influential role in fibrogenesis in many tissues, such as the kidney, liver heart and lung, and increases myofibroblasts and ECM. Similar to EMT, EndoMT defines as a complex biological process in which ECs lose their endothelial markers, adhesion and apical-basal polarity and transit into mesenchymal cell type under certain conditions, leading to vascular rarefaction, organ fibrosis and dysfunction (Zeisberg et al., 2008; Dejana et al., 2017).

Podocytes, as special glomerular epithelial cells, can obtain a mesenchymal property in a high-glucose condition, which is also called EMT (Yamaguchi et al., 2009). In addition to the aforementioned cells, PECs have been receiving great attention as progenitor cells of podocytes. Activated PECs are one of the possible sources of podocytes through phenotypic transitions at the vascular pole and increase the expression and secretion of ECM proteins in DN (Appel et al., 2009).

## 4 Cellular transdifferentiation in the glomerulus

### 4.1 Podocyte-mesenchymal transition in DN

**FIGURE 1:**
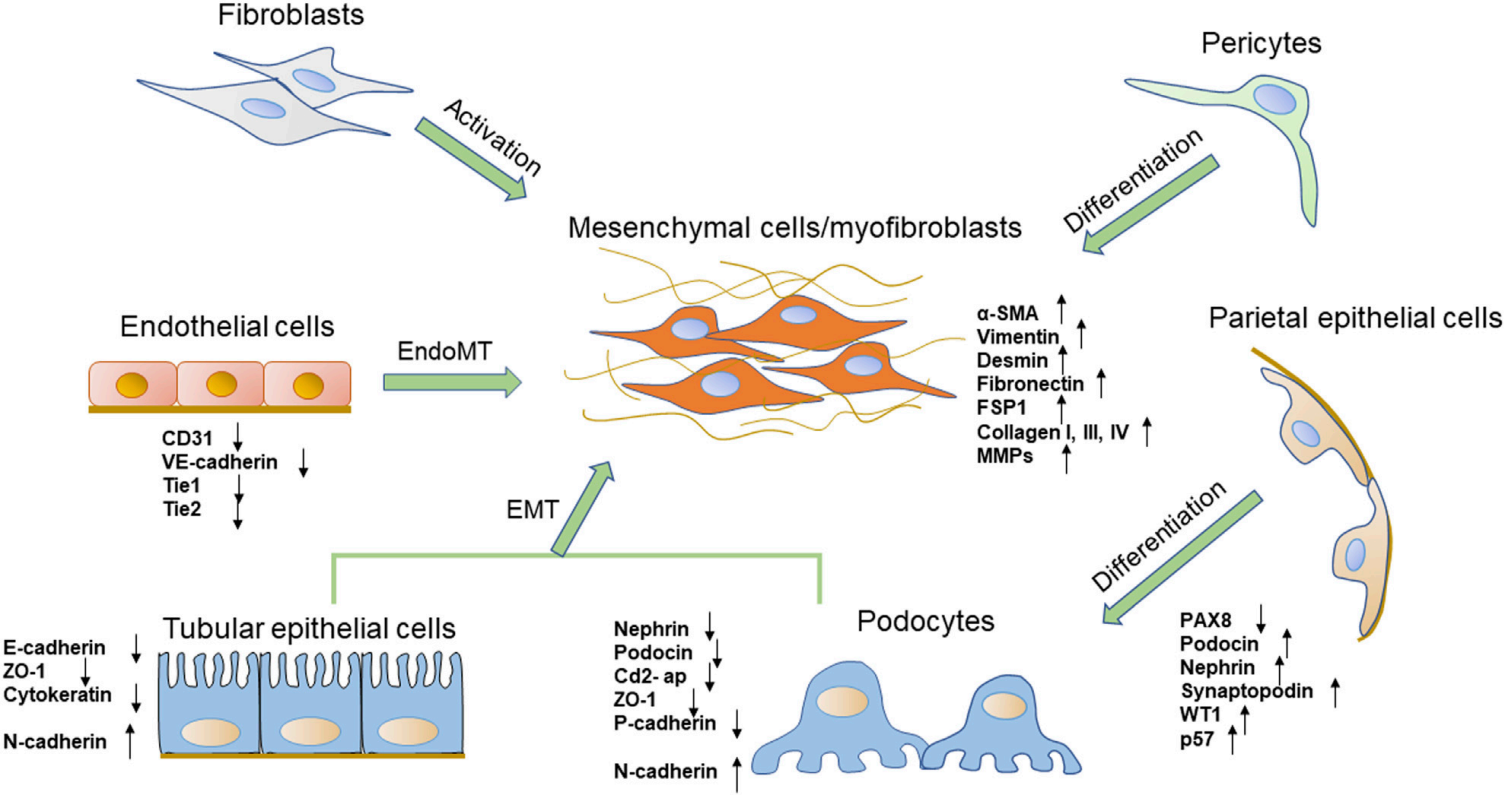
The phenotypic transitions of several types of renal cells and the expressions of markers. The origin of the myofibroblasts includes several types of cells, such as fibroblasts, pericytes, tubular epithelial cells, and endothelial cells. Podocytes also obtain a mesenchymal property through mesenchymal transition. PECs are one of the possible sources of podocytes.

Glomerular visceral epithelial cells, also called podocytes, are terminally differentiated cells that circle the basement membrane of the glomerular capillary. As terminal differentiation cells, podocytes cannot regenerate when they suffer from injury (Faul et al., 2007). Podocyte foot processes interdigitate with each other as well as with neighboring podocytes to form a filtration slit called a slit diaphragm (Greka and Mundel, 2012). Several proteins associated with the slit diaphragm, such as nephrin, podocin, CD2-associated protein (Cd2-ap), and zonula occludens-1 (ZO-1), are of critical importance in establishing the glomerular filtration barrier (Zhou and Liu, 2015). As the most important component of the renal filtration barrier, podocytes are vulnerable to hyperglycemia and play a crucial role in pathological mechanisms underlying DN. Studies *in vivo and in vitro* found that podocytes exposed to high glucose concentration could undergo mesenchymal transition, which aggravated podocytes’ structure and function injury (Yamaguchi et al., 2009; Zhang et al., 2012). In this process, podocytes appear to lose their epithelial polarity, alteration of the cell-cell junctions represented by a loss of the slit diaphragm, and rearrangement of the actin cytoskeleton, which finally causes foot process effacement. Podocytes lose specific markers expression of nephrin, podocin, ZO-1 and P-cadherin (Shankland, 2006) (Figure 1). It has been established that mutations of these proteins lead to foot process effacement and podocyte dysfunction resulting in defective glomerular filtration along with the increase in proteinuria. By contrast, the expression levels of mesenchymal cell marker proteins, such as fibroblast-specific protein-1 (FSP-1), α-SMA, desmin, matrix metalloproteinase 9 (MMP9), and expression of the main transcription factors: Snail1 and Snail2 are increased (Yamaguchi et al., 2009). These changes lead to podocyte dysfunction, filtration barrier damage, and protein leakage, thereby ultimately promoting glomerular sclerosis and the progress of DN (Yamaguchi et al., 2009). These phenotypic phenomena are consistent with the process of EMT (Liu, 2010).

However, it should be noted that the phenotype transition of podocytes does not satisfy all the criteria for EMT. First of all, one of the criteria for EMT is a cadherin switch from E-cadherin to N-cadherin. But podocytes express P-cadherin instead of E-cadherin and undergo a cadherin switch from P-cadherin to N-cadherin, which precludes them from matching this definition of EMT. Besides, no evidence showed that podocytes expressed markers of EMT like heat shock protein 47 (HSP47) and discoidin or domain receptor 2 (DDR2) in the literature (Zeisberg and Neilson, 2009; Galichon et al., 2011; May et al., 2014; Kim et al., 2017).

### 4.2 PECs transdifferentiation in DN

Recent studies have suggested that PECs are directly involved in the pathogenesis of certain glomerular diseases. PECs, which line the inner surface of Bowman’s capsule, resemble squamous epithelial cells and form primary cilia. PECs and podocytes arise from the same ancestral cell but unlike podocytes, PECs maintain the ability to proliferate under physiological conditions (Shankland et al., 2014). They act as a selective permeability barrier to the urinary filtrate and prevent proteins in the glomerular ultrafiltrate in the Bowman’s space from entering the extraglomerular space (Ohse et al., 2009). Tight junctions are present between adjacent PECs in normal glomeruli, The junction proteins including claudin-1, ZO-1 and occludin form an impermeable barrier. PECs could increase the potential for proliferation, migration, production of extracellular matrix and chemokines and cytokines, and expression of certain markers (Holderied et al., 2015; Lazareth et al., 2020). Researchers have found that PECs serve as potential reparative and regenerative precursors for podocytes when podocytes are lost (Appel et al., 2009; Kietzmann et al., 2015). PECs proliferate with aging and differentiate into podocytes to supplement podocyte deletion. They could co-express PEC and podocyte-specific proteins, acquire ultrastructural features of podocytes and migrate from Bowman’s capsule to the capillary tuft (Kaverina et al., 2020). Activated PECs can express specific podocytes markers, like podocin, nephrin, synaptopodin, Wilms’ tumor 1 (WT1), p57 and they still express the activation marker for PECs, CD44, but no longer express the PEC marker paired box 8 (PAX8) and lose the capacity to proliferate (Figure 1) (Schulte et al., 2014; Kaverina et al., 2020). Several studies have shown the importance of the activation of PECs as progenitor cells to regenerate podocytes in human and mouse DN models (Pichaiwong et al., 2013; Andeen et al., 2015). Activated and proliferated PECs were observed in diabetic rats and were positively correlated with the extent of proteinuria and podocyte loss (Zhao et al., 2011). In another DN model, authors have identified that glomerular PECs could function as a possible source of regenerating podocyte progenitor for podocyte restoration, which may reverse or prevent DN (Pichaiwong et al., 2013; Luna-Antonio et al., 2017). The mechanism of PECs activation and PECs transition is rarely understood. Notch signaling pathway and extracellular signal-regulated kinase (ERK) signaling pathway may involve in the transition from PECs to podocytes (Sweetwyne and Susztak, 2013; Urushihara et al., 2020). Wnt/β-catenin signaling pathway, which is a widely recognized pathway that mediates cell differentiation, is also likely involved in PECs transition (Ni et al., 2021). However, more researches need to be employed for a better understanding of the potential role of activated PECs in DN.

## 5 Cellular transdifferentiation in the tubulointerstitium

### 5.1 EMT of TECs in DN

EMT of renal TECs is one of the major pathogeneses of renal interstitial fibrosis. TECs are postulated to contribute to the phenotypic change in myofibroblasts through the process of EMT (Liu, 2010; Galichon and Hertig, 2011). During that process, injured TECs are activated and lose their epithelial phenotype and acquire new characteristic features of mesenchymal cells. The processes are summarized as four key events. First, TECs lose epithelial cell junctions and cell polarity. Then, the epithelial genes such as E-cadherin, ZO-1 are suppressed, and mesenchymal markers including α-SMA, vimentin, FSP1, type I collagen and FN are activated (Liu et al., 2018a) (Figure 1). E-cadherin plays a crucial role in maintaining the structural integrity of renal epithelia and its polarization, and the downregulation of the E-cadherin expression is an important change in EMT (Kalluri and Neilson, 2003). Next, the cytoskeletal architecture reorganizes and changes cellular morphology. Finally, they obtain the ability to secrete factors like matrix metalloproteinases (MMPs) to disrupt the basement membrane and enhanced invasive behavior (Yang and Liu, 2001; Lamouille et al., 2014).

TECs and myofibroblasts are normally separated by a tubular basement membrane, which prevents contact between TECs and interstitial matrix components (Fan et al., 1999). The completion of an EMT is signaled by the degradation of the underlying basement membrane and TECs increase motility, through which they cross the barrier and migrates into the interstitium (Kalluri and Weinberg, 2009; Das et al., 2019). However, studies have questioned the role of EMT in renal fibrosis based on evidence that epithelial cells actively cross the tubular basement membrane and could not find a direct contribution of epithelial cells to the myofibroblast population (Humphreys et al., 2010; LeBleu et al., 2013; Lovisa et al., 2016). Recent studies offered new insights into the potential role of tubular EMT in renal fibrosis. They found a large population of differentiated TECs co-expressed epithelial and mesenchymal markers and remained associated with their basement membrane after renal injury (LeBleu et al., 2013; Lovisa et al., 2016). This type of renal epithelial cell is considered to have undergone incomplete EMT called partial EMT. Partial EMT is in agreement with earlier studies (Kriz et al., 2011; Hajarnis et al., 2018). Although TECs remain attached to the basement membrane during fibrosis rather than cross the basement membrane to join the myofibroblast pool, evidence has demonstrated partial EMT is sufficient to induce tubular function impairment. The expression of TECs fundamental proteins such as E-cadherin and ZO-1 reduces, which impairs TECs polarity and junction, and by triggering cell cycle arrest impairing the repair of the damaged tissue. Partial EMT also modifies the epithelial secretome profile thereby promoting the secretion of profibrogenic cytokines and fibrosis (Grande et al., 2015; Lovisa et al., 2015).

### 5.2 Fibroblast differentiation in DN

Fibroblasts, the main cells of the renal interstitial space, are important for maintaining the structural integrity of kidneys by maintaining constituent ECM. About 50% of myofibroblasts in unilateral ureteral obstruction (UUO) are shown to be originated from resident fibroblasts *via* proliferation (LeBleu et al., 2013). Aberrant activation of fibroblasts and transformation into myofibroblasts contribute to progressive fibrotic events, which are the main cellular source of the ECM and contribute significantly to the pathogenesis of kidney fibrosis (Chen et al., 2019). Activated fibroblasts acquire smooth muscle features including expression of desmin and α-SMA (Figure 1) (Gabbiani, 2003; Hinz et al., 2007). Fibroblasts are activated by inflammatory cytokines, growth factors, hypoxia and mechanical forces to proliferate and produce ECM components like collagen I, III, IV and fibronectin (Kessler et al., 2001). Injured TECs are potent producers of growth factors and cytokines such as transforming growth factor-β (TGF-β), platelet-derived growth factor (PDGF), hedgehog, and Wnt ligands, which could cause fibroblasts to transform into myofibroblasts (Dussaule et al., 2011; Meran and Steadman, 2011). TGF-β-mediated fibroblast activation is thought to be the major driver of kidney fibrosis in DN (Desmouliere et al., 1993; Gerrits et al., 2020).

## 6 Cellular transdifferentiation from microvessels

ECs and pericytes are the two main cellular constituents in renal microvessels. Both are of great interest for research on phenotypic transitions in the development of DN.

### 6.1 EndoMT in DN

The kidney is a highly vascularized organ, characterized by a remarkable diversity of EC populations (Verma and Molitoris, 2015). The glomerular ECs contribute to the glomerular filtration barrier and support podocyte structure, whereas ECs of the microvasculature around kidney tubules contribute to tubular reabsorption (Jourde-Chiche et al., 2019). When renal ECs are injured, the production of nitric oxide (NO) decreases and vasodilation function is impaired, which decreases renal blood flow and initiation of EndoMT (Basile et al., 2018). EndoMT, which happens in tubulointerstitial vessels as well as glomerular ECs, appears to play a significant role in the development and progression of DN (Lu et al., 2021). In 2008, a study by Zeisberg et al. (2008) firstly identified the crucial role of EndoMT in DN. Studies demonstrated a remarkable ratio of myofibroblasts that could co-expressed endothelial markers and myofibroblast markers in streptozotocin (STZ)-induced DN, indicating the endothelial origin of myofibroblasts. They declared that the 30–50% activated fibroblast cells were derived from ECs. Another study investigated the role of EndoMT in mice and demonstrated that 10–24% of renal interstitial myofibroblasts emerged *via* EndoMT in STZ-induced DN mice (Song et al., 2019). However, a study in 2013 revealed that only 10% of myofibroblasts were of endothelial origin following kidney injury (LeBleu et al., 2013). EndoMT is also observed co-expression of endothelial and mesenchymal markers in glomeruli of DN patients. During EndoMT, renal ECs gradually lose their specific endothelial cell markers, such as platelet endothelial cell adhesion molecule-1 (PECAM-1/CD31), VE-cadherin, tyrosine kinase with immunoglobulin like and EGF like domains 1 (Tie1) and TEK Receptor Tyrosine Kinase (Tie2), while acquiring mesenchymal or myofibroblastic phenotype genes and proteins, such as α-SMA, N-cadherin, FSP-1 and increase the production of ECM proteins (Figure 1) (Sanchez-Duffhues et al., 2018). Meanwhile, ECs lose endothelial characteristics such as increased vascular permeability, polarization and cell-cell connections. Thus, these cells get a high migration potential and promote the development of renal fibrosis (Kizu et al., 2009). Dysfunctional glomerular ECs could communicate with adjacent podocytes and mesangial cells *via* secreting effectors, which aggravates glomerular injury and creates a vicious cycle that leads to proteinuria in diabetic patients (Fu et al., 2015).

### 6.2 Pericytes differentiation in DN

Pericytes are present in the microvasculature, at terminal arterioles, capillaries and post-capillary venules. They share a common basement membrane with ECs (Stratman et al., 2009; van Dijk et al., 2015). In the kidney, pericytes are located in the tubular system called peritubular pericytes, and in renal glomeruli called mesangial cells (also described as pericyte-like cells) (Schlondorff, 1987; Geevarghese and Herman, 2014). Mesangial cells play an important role in supporting glomerular capillary network. They display pericyte functions by regulating glomerular blood flow and controlling glomerular filtration rate due to their contractility (Kramann and Humphreys, 2014). Like pericytes, mesangial cells express PDGFR-β and generate mesangial basement membrane molecules, such as collagen IV and FN (Isono et al., 2002). PDGFR-β has a critical role in pericyte activation, proliferation, differentiation and communication with ECs (Leveen et al., 1994). According to experimental and clinical studies, mesangial cells are considered to be an important precursor cell type of myofibroblasts in DN (Simonson, 2007) (Figure 1). High glucose enhances mesangial cells to undergo proliferation and alters the normal phenotype to express α-SMA and other growth factors, causing mesangial matrix expansion (Koya et al., 2000; Isono et al., 2002).

In the tubulointerstitial system, peritubular pericytes are essential for peritubular capillary integrity as well as tubular integrity (Kida et al., 2013). Under pathological conditions, peritubular pericytes are activated and are the key precursor of myofibroblasts. Pericytes subsequently migrate from the peritubular capillaries into the interstitium and differentiation into myofibroblasts resulting in scar formation, progressive fibrosis and deterioration of renal function (Lin et al., 2008; Humphreys et al., 2010). Meanwhile, pericytes differentiation into myofibroblasts also loses vessel stabilizing abilities leading to peritubular capillary disintegration and rarefaction after the injury (Schrimpf et al., 2012). Pericytes also synthesize erythropoietin (EPO) in normal kidneys, while myofibroblasts lose the ability to produce EPO, leading to renal anemia and thereby enhanced tissue hypoxia (Chang et al., 2016; Souma et al., 2016). Studies showed inhibition of PDGFR signaling prevented the proliferation and differentiation of pericytes and reduced interstitial kidney fibrosis. Therefore, targeting pericytes may provide a novel strategy to prevent kidney fibrosis (Chen et al., 2011). However, the studies involved in the role of pericytes in DN are limited. Future studies should continue identifying the specific contribution of pericytes to DN and renal fibrosis (Hayes, 2019).

## 7 Molecular pathways mediating phenotypic transitions in DN

**FIGURE 2:**
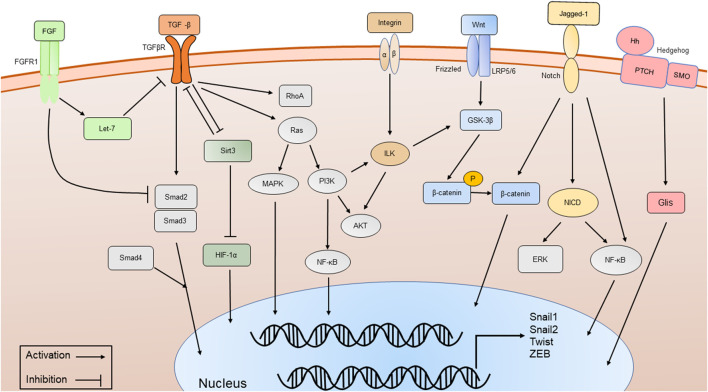
The major signaling pathways involved in phenotypic transitions, including TGF-β/Smad-dependent and Smad-independent signal transduction pathways, Wnt/β-catenin signaling pathway, Integrin/ILK signaling pathway, Notch signaling pathway, Sirt3 signaling pathway, FGFR1 and Hedgehog signaling pathway.

Various molecular pathways in phenotypic transitions in renal diseases have been studied. But in DN, research is still limited (Table 1). TGF-β plays a significant role in phenotypic transitions of renal cells (Figure 2). In elevated glucose levels, TGF-β increases in podocytes, TECs, ECs and pericytes to induce mesenchymal transitions *via* several SMAD family member (Smad)-dependent and Smad-independent signal transduction pathways (Hills and Squires, 2010; Xie et al., 2015; Wu et al., 2017; Dong et al., 2019a). In the classic TGF-β/Smad signaling pathway, TGF-β binds to its receptor TGF-βR1, and then Smad2/3 is phosphorylated and combines with Smad4 to form a Smad complex, followed by translocation into the nucleus and activation of transcription of pro-fibrotic genes like α-SMA, connective tissue growth factor (CTGF), MMP-9 and Snail. Several research studies have been conducted focusing on the regulation of TGF-β/Smad signaling pathway (Lan, 2012). The negative regulator ski-related protein N (SnoN), Sirtuin 6 (Sirt6) prevented nuclear translocation or deacetylated Smad3. Many micro-RNA like mir-17, mir-30c and circRNA could inhibit TGF-β/Smad signaling pathway (Zhao et al., 2017; Srivastava et al., 2019; Fu et al., 2021). Like EMT, the TGF-β/Smad-dependent signaling pathway has also become a hot topic in EndoMT research (Li et al., 2010). TGF-β/Smad-dependent signaling pathway induces specific target genes, including Snail1, Snail2 and Twist, leading to the initiation of EndoMT (Song et al., 2019; Gwon et al., 2020). Inhibition of EndoMT by blockade of TGF-β/Smad signaling could reduce renal fibrosis, and retard the progression of nephropathy in STZ-induced DN (Shi et al., 2020). However, Smad-independent pathways of TGF-β signaling involved in phenotypic transitions include ras homolog gene family member A (RhoA) (Bijkerk et al., 2012; Zhang et al., 2013), extracellular signal-regulated kinase (ERK) (Zhou et al., 2010; Li et al., 2017a; Dong et al., 2019b), p38 mitogen-activated protein kinase (MAPK) (Bakin et al., 2002; Li et al., 2017a; Deng et al., 2020; Li et al., 2022), and phopshatidylinositol 3 kinase (PI3K)/protein kinase B (Akt) (Lee and Han, 2010; Lu et al., 2019), Ras (Su et al., 2020), integrin-linked kinase (ILK) (Du et al., 2016; Qi et al., 2018; Li et al., 2019a), nuclear factor kappa B (NF-κB) (Li et al., 2012; Wei et al., 2021) and β-catenin (Wang et al., 2011). For example, ERK could disassemble adherens junctions and promote cell motility by controlling the expression of E-cadherin and ZO-1 (Xie et al., 2004). C-reactive protein (CRP) promoted EMT only in the presence of TGF-β *via* ERK1/2 signaling (Zhang et al., 2019). Heparanase could also promote EndoMT *via* activating ERK signaling (Chang et al., 2022). Methyltransferase Like 14 (METTL14) regulated PI3K/Akt signaling pathway *via* phosphatase and tensin homolog (PTEN), which affected EMT of renal tubular cells in DN (Xu et al., 2021). The Smad-dependent and Smad-independent pathways can coordinate in the regulation of phenotypic transitions.

**TABLE 1:** The major signaling pathways involved in phenotypic transitions in DN.

Signaling pathways		Phenotypic transitions	References
TGF-β	Smad	EMT, EndoMT and mesangial cells	Hills and Squires. (2010); Xie et al. (2015); Wu et al. (2017); Dong et al. (2019a); Li et al. (2010); Zhao et al. (2017); Shi et al. (2020)
	ERK	EMT, EndoMT	Dong et al. (2019b); Du et al. (2016); Zhang et al. (2019); Chang et al. (2022)
	p38/MAPK	EMT	Li et al. (2017a); Li et al. (2022)
	PI3K/AKT	EMT	Lee and Han, (2010); Lu et al. (2019); Xu et al. (2021)
	ILK	EMT	Du et al. (2016)
	NF-κB	EMT	Wei et al. (2021)
β-catenin	GSK-3β/Wnt	EMT, EndoMT and mesangial cells	Guo et al. (2014); Li et al. (2015); Dai et al. (2017); Wang et al. (2020); Xie et al. (2020); Zhang et al. (2020); Liu et al. (2021)
	CRP	EMT	Zhang et al. (2019)
	CTGF	EMT	Dai et al. (2017); Dai et al. (2016)
	Rac1/PAK1	EMT	Lv et al. (2013)
	NF-κB	EMT	Ghiggeri et al. (2013)
	VDR	EMT	Guo et al. (2014)
ILK	Integrins/ILK	EMT and mesangial cells	Li et al. (2003); Dai et al. (2012); Niu et al. (2014); Kang et al. (2010)
	Akt	EMT	Zhang et al. (2018b)
	TGF-β	EMT	Zhang et al. (2018b)
Sirt3	HIF-1α	EMT	Li et al. (2020a)
	TGF-β/Smad	EndoMT	Srivastava et al. (2021)
FGFR	FGFR1	EMT, EndoMT	Li et al. (2020b)
	TGF-β	EndoMT	Chen et al. (2014)
Notch	Notch	EMT, EndoMT	Tian et al. (2018); Nishad et al. (2019); Nishad et al. (2020); Liu et al. (2018b)
Hedgehog	Hedgehog	EndoMT	Zhao et al. (2018)
	TGF-β	EMT	Li et al. (2019b)

Another key molecule is named β-catenin (Figure 2). Wnt/β-catenin signaling pathway expression is increased in podocytes, TECs and ECs of hyperglycemic patients, as well as mouse models and plays a critical role in integrating cell differentiation (Lee and Han, 2010; Li et al., 2015; Dai et al., 2017). β-catenin assembles in the cytoplasm and translocates to the nucleus, regulates the expression of Wnt target genes and enhances myofibrogenesis. Suppressing the Wnt/β-catenin signaling pathway has been shown to restrain renal fibrosis. Many compounds like Panax Notoginseng and Astragaloside IV have been proven to exert renal protection by inhibiting the Wnt/β-catenin signaling pathways in STZ-induced animal models (Wang et al., 2020; Xie et al., 2020; Zhang et al., 2020). Glycogen synthase kinase 3β (GSK-3β) showed an increased expression and promoted the EMT of podocytes in high glucose by classical GSK- 3β/Wnt/β-catenin (Guo et al., 2014). CRP, CTGF and RAS-related C3 botulinum toxin substrate 1 (Rac1)/p21-activated kinase 1 (PAK1), NF-κB signaling could promote podocyte EMT by regulating the nuclear translocation of β-catenin under a high-glucose condition (Ghiggeri et al., 2013; Lv et al., 2013; Dai et al., 2016; Zhang et al., 2019). In contrast, Caudal-type homeobox transcription factor 2 (CDX2) could improve renal EMT during DN by suppressing the Wnt/β-catenin signaling pathway (Liu et al., 2021). Vitamin D receptor (VDR) competed with β-catenin in combination with transcription factor 4 (TCF4) and inhibited the activity of β-catenin. In the high-glucose environment, VDR decreased and aggravated EMT (Guo et al., 2014).

Integrin-linked kinase (ILK) is an intracellular serine/threonine kinase that interacts with integrins and activates several downstream effectors such as Akt and GSK-3β, which are involved in the regulation of cell adhesion, cell shape changes, and deposition of the ECM[(Naves et al., 2012; Zhang et al., 2018b)]. Integrins/ILK signaling pathway (Figure 2) has been proven to induce EMT in TECs and podocytes (Li et al., 2003; Dai et al., 2012; Niu et al., 2014). As a protein kinase, ILK directly phosphorylates several physiologically important downstream effector kinases including p38 MAPK, Akt and GSK-3β, leading to the stabilization and accumulation of β-catenin (Hannigan et al., 2005). The inhibition of ILK ameliorated proteinuria or attenuates EMT (Li et al., 2009; Kang et al., 2010). The effect of ILK also has been identified as partially regulated by TGF-β/Smad signaling pathway. Inhibition of ILK could block podocytes EMT induced by TGF-β (Li et al., 2003). Hesperetin produced protective effects in podocyte EMT possibly by suppressing TGF-β/ILK/Akt signaling in DN (Zhang et al., 2018b).

Mitochondrial deacetylase sirtuin 3 (Sirt3) and fibroblast growth factor receptors (FGFRs) may be the key signaling pathways that inhibit EMT and EndoMT (Figure 2) (Li et al., 2017b; Li et al., 2020a; Srivastava et al., 2021). Sirt3 deficiency induces hypoxia inducible factor 1α (HIF-1α) overaccumulation, which results in kidney fibrosis *via* induction of the EMT (Higgins et al., 2007; Sun et al., 2009). Sodium-glucose Cotransporter-2 (SGLT2) inhibitor empagliflozin has direct effects on tubule metabolic pathways associated with the inhibition of aberrant glycolysis and restoration of Sirt3 expression, resulting in suppression of the EMT (Li et al., 2020a). Studies showed endothelial Sirt3 pathway regulated glucose and lipid metabolism and related EndoMT processes by maintaining control of TGF-β/Smad3 signaling in the kidney of diabetic mice. Sirt3 knockout in mice resulted in increased levels of EndoMT and reactive oxygen species (ROS), and promoted renal dysfunction, while in Sirt3 knock-in EC-specific transgenic mice, renal fibrosis and EndoMT, as well as oxidative stress, were ameliorated (Srivastava et al., 2021). Studies observed that fibroblast growth factors (FGF)/FGFR1 signaling was downregulated in the kidney of DN (Baelde et al., 2004; Liang et al., 2018). FGF/FGFR1 signaling has been confirmed to suppress TGF-β signaling in ECs (Chen et al., 2014). Diabetic FGFR1 knockout mice have significantly higher EndoMT and EMT levels and severe organ fibrosis (Li et al., 2020b).

Notch signaling has been shown to mediate EMT and EndoMT in DN (Asfahani et al., 2018; Tian et al., 2018; Nishad et al., 2019). Notch receptors on the cell surface bind various ligands, including Jagged-1, resulting in the cleavage of Notch receptor by proteases and notch intracellular domain (NICD) translocates to the nucleus to regulate the expression of transcription factors like Snails (Hayward et al., 2008). Growth hormone induced Notch signaling in podocytes and contributed to proteinuria through EMT as well as renal fibrosis (Nishad et al., 2019). Advanced glycation end-products (AGEs) could activate Notch signaling in podocytes and provoke EMT (Nishad et al., 2020). Notch also indirectly regulates EMT through various signaling pathways, including NF-κB and β-catenin, and through the action of various regulatory miRNAs (Yang et al., 2011) (Figure 2). Overexpression of Notch in ECs resulted in the loss of VE-cadherin and subsequent EndoMT (Timmerman et al., 2004). Over-expression of NUMB endocytic adaptor protein (Numb) inhibited the expression of Notch signaling within EMT of DN (Liu et al., 2018b). Notch signaling activation mediated TGF-β-induced EMT in both human and rat TECs in cultured (Bielesz et al., 2010). MMP-9 played an important role in TGF-β-induced EndoMT *via* upregulation of Notch signaling in glomerular ECs (Zhao et al., 2016).

Hedgehog signaling pathway (HH) is a well-known pathway in EMT (Gonzalez and Medici, 2014). Members of the Hedgehog family contain sonic hedgehog (Shh), Indian hedgehog (Ihh), and desert hedgehog (Dhh). The HH pathway consists of two transmembrane proteins, patched (Ptch) and smoothened (Smo) (Figure 2). Active Smo promotes transcription factors GLI family zinc finger (Glis) nuclear translocation. Mesenchymal stem cells reversed the renal injury and EMT process by blocking NF-κB/hedgehog pathways (Xiao et al., 2020). Hyperglycemia elevated renal hedgehog-interacting protein (Hhip) gene expression subsequently activated the TGFβ1/Smad2/3 cascade and promotes EndoMT (Zhao et al., 2018; Zhao et al., 2021). PTEN could induce partial EMT through Hedgehog signaling pathway in DN mice (Li et al., 2019b).

## 8 Conclusion and perspective

DN is closely related to renal function loss in patients and is one of the major causes of end stage renal disease throughout the world. Renal cellular phenotypic transitions have become one of the most fascinating topics in the studies of organ fibrosis in recent years. In this review, we discussed the cellular phenotypic transitions in the kidney. TECs, ECs, fibroblasts and pericytes are important precursors of myofibroblasts. Podocytes also undergo similar phenotypic changes to EMT under high glucose conditions, which promote the development of glomerulosclerosis. PECs proliferate and differentiate into podocytes when podocyte loss occurs during injury. Most of the known molecular mechanisms and downstream interactions between TGF-β/Smad classic pathway and multiple other pathways of phenotypic transitions in DN are summarized. However, the understanding of some processes like pericytes to myofibroblasts, PECs to podocytes, or EndoMT in DN has just begun. Various molecular mechanisms remain unclear in DN. More work in the future should focus on deeply exploring the role of these cells and strive to find potential new targets for the treatment of renal fibrotic diseases, which is of great significance for the development of DN treatment.

## Glossary

DM: diabetes mellitus
DN: diabetic nephropathy
GFR: glomerular filtration rate
ECM: extracellular matrix
PECs: parietal epithelial cells;
ER: endoplasmic reticulum
FN: fibronectin
GBM: glomerular basement membrane
α-SMA: α-smooth muscle actin
PDGFR: platelet-derived growth factor receptor
EndoMT: endothelial-mesenchymal transition
EMT: epithelial-mesenchymal transition
Snail: Snail family transcriptional repressor
Twist: Twist family
BHLH: Transcription Factor
ZEB: Zinc finger E-Box binding homeobox
ECs: endothelial cells
Cd2-ap: CD2-associated protein
ZO-1: zonula occludens-1
MMP9: matrix metalloproteinase 9
FSP1: fibroblast-specific protein-1
HSP47: heat shock protein 47
DDR2: discoidin or domain receptor 2
WT1: Wilms’ tumor 1
PAX8: paired Box 8
ERK: extracellular signal-regulated kinase;
MMPs: matrix metalloproteinases
TECs: tubular epithelial cells
UUO: unilateral ureteral obstruction
TGF-β: transforming growth factor β
NO: nitric oxide
PECAM-1/CD31: platelet endothelial cell adhesion molecule-1
Tie1: tyrosine kinase with immunoglobulin like and EGF like domains 1
Tie2: TEK receptor tyrosine kinase
EPO: erythropoietin
Smad: SMAD family member
CTGF: connective tissue growth factor
SnoN: ski-related protein N
Sirt6: Sirtuin 6
RhoA: The Ras homolog gene family, member A
MAPK: p38 mitogen-activated protein kinase
PI3K: phosphoinositide 3-kinases
AKT: protein kinase B
ILK: integrin-linked kinase
NF-κB: nuclear factor kappa B
CRP: C-reactive protein
METTL14: methyltransferase Like 14
PTEN: phosphatase and tensin homolog
GSK-3β: glycogen synthase kinase 3β
Rac1: RAS-related C3 botulinum toxin substrate 1
PAK1: p21-activated kinase 1
CDX2: caudal-type homeobox transcription factor 2
VDR: vitamin D receptor
TCF4: transcription Factor 4
Sirt3: mitochondrial deacetylase sirtuin 3
FGFRs: fibroblast growth factor receptors
HIF-1α: hypoxia inducible factor 1
SGLT2: sodium-glucose cotransporter-2
ROS: reactive oxygen species
FGF: fibroblast growth factors
NICD: notch intracellular domain
AGEs: advanced glycation end-products
Numb: NUMB endocytic adaptor protein
Glis: GLI family zinc finger
Hhip: hedgehog-interacting protein
